# Non-essentiality of canonical cell division genes in the planctomycete *Planctopirus limnophila*

**DOI:** 10.1038/s41598-019-56978-8

**Published:** 2020-01-09

**Authors:** Elena Rivas-Marin, Stijn H. Peeters, Laura Claret Fernández, Christian Jogler, Laura van Niftrik, Sandra Wiegand, Damien P. Devos

**Affiliations:** 1 Centro Andaluz de Biología del Desarrollo (CABD)-CSIC, Pablo de Olavide University, Seville, Spain; 2 Department of Microbiology, IWWR, Faculty of Science, Radboud University, Nijmegen, The Netherlands; 3 Institute of Microbiology, Department of Microbial Interactions, Friedrich-Schiller-Universität Jena, Jena, Germany

**Keywords:** Cellular microbiology, Bacterial genetics

## Abstract

Most bacteria divide by binary fission using an FtsZ-based mechanism that relies on a multi-protein complex, the divisome. In the majority of non-spherical bacteria another multi-protein complex, the elongasome, is also required for the maintenance of cell shape. Components of these multi-protein assemblies are conserved and essential in most bacteria. Here, we provide evidence that at least three proteins of these two complexes are not essential in the FtsZ-less ovoid planctomycete bacterium *Planctopirus limnophila* which divides by budding. We attempted to construct *P. limnophila* knock-out mutants of the genes coding for the divisome proteins FtsI, FtsK, FtsW and the elongasome protein MreB. Surprisingly, *ftsI*, *ftsW* and *mreB* could be deleted without affecting the growth rate. On the other hand, the conserved *ftsK* appeared to be essential in this bacterium. In conclusion, the canonical bacterial cell division machinery is not essential in *P*. *limnophila* and this bacterium divides via budding using an unknown mechanism.

## Introduction

In all life forms, cell division is a highly conserved and regulated process. Current knowledge about bacterial cell division is largely based on a few model organisms, such as *Escherichia coli*, *Bacillus subtilis* and *Caulobacter crescentus*. Most bacteria divide by binary fission, a widely conserved mechanism based on the interaction between the FtsZ protein and the peptidoglycan (PG) biosynthesis machinery (PG synthesis reviewed in^1^). FtsZ is an almost universally conserved bacterial cytoskeletal element, homologous to the eukaryotic tubulin^2–4^. This bacterial cytoskeleton is the central member of the cell division machinery, the divisome, a dynamic protein complex composed of more than 20 conserved proteins that assembles at mid-cell^5,6^. FtsZ filaments were previously believed to form a continuous ring (the Z-ring), closing concentrically during division and splitting both cells. However, the continuity of this ring is currently questioned^7^ and recent data suggest that FtsZ forms a single-layered structure at a distance of several nanometers from the inner membrane^8^. The FtsZ filaments treadmill on the inner face of the cytoplasmic membrane and interact with the PG synthases^9,10^. However, the precise nature of the interaction between the Z-ring protofilaments and their physiological significance remains a mystery^11^. Whatever its precise organization, this structure determines the future division site and serves as a scaffold to hierarchically recruit the remaining division players^8^. Additionally, the ring participates in chromosome segregation^12^.

The Z-ring is a quasi-universal element of cytokinesis in bacteria that possess PG and divide by binary fission. There are however a few prokaryotic exceptions to this FtsZ-dominated division mode. First of all, FtsZ is not the dominant division machinery in *Archaea* where at least three distinct division systems are found based on FtsZ, ESCRT-III homologs, or actin-related proteins^13,14^.

Secondly, the gene coding for FtsZ is also absent from the genomes of a limited number of bacteria. These include pathogenic strains from the *Tenericutes*, the *Chlamydiae*, and some symbionts such as the gammaproteobacteria “*Candidatus* Ruthia magnifica”*, “Candidatus* Vesicomyosocius okutanii HA” and *“Candidatus* Carsonella ruddii”, the alphaproteobacterium “*Candidatus* Hodgkinia cicadicola”, and the bacteroidete “*Candidatus* Sulcia muelleri”^15^. In most of these cases, the loss of *ftsZ* might be related to the extreme genome reduction associated with their parasitic or endosymbiotic lifestyle. At least in the case of *Wolbachia*, a genus of *Alphaproteobacteria*, it has been shown that the *ftsZ* gene has been transferred to the *Drosophila* host genome^16^. In addition, FtsZ is not found in the *Planctomycetes* phylum, which is composed of free-living bacteria^17,18^.

Among the proteins forming the divisome, there are PG synthases and hydrolases that remodel the PG, DNA translocases which establish communication between chromosome replication-segregation and cell division machineries, and proteins that coordinate inner and outer membrane constriction^19,20^. Twelve of the proteins that compose the divisome are described to be essential and conserved across bacteria: FtsZ, FtsA, ZipA, FtsE, FtsX, FtsK, FtsQ, FtsL, FtsB, FtsW, FtsI and FtsN^21,22^.

Whereas during division the PG is synthesized at the new cell poles^23^, during cell elongation, the PG precursor, lipid II, is incorporated into the previously existing cylindrical part of the cell wall^24^. This lateral cell wall synthesis is performed by the elongasome, a protein complex formed by MreBCD, RodA, RodZ, PBP1A, PBP2 as well as MurF, MurG and MraY^25,26^. The complex is guided by the actin-like protein MreB^27–29^, which is essential for cell elongation and maintenance of the cell shape. MreB is extremely conserved, being found in almost all non-spherical bacteria except the ones exhibiting polar growth. Some examples of MreB-lacking rods are members of the *Actinobacteria* and *Alphaproteobacteria* (*Rhizobium* and *Agrobacterium*)^30,31^. In contrast, other Gram-positive rod-shaped bacteria, such as *B. subtilis*, have multiple *mreB* copies^32^.

Apart from binary fission, other cell division mechanisms, such as multiple intracellular offspring, multiple offspring by binary fission, multiple fission or budding, have been reported in some members of *Cyanobacteria*, *Firmicutes*, *Planctomycetes* and the prosthecate proteobacteria^33,34^. Recent studies also revealed asymmetric division in *Chlamydia trachomatis*^35,36^. Bacteria belonging to the PVC superphylum (*Planctomycetes-Verrucomicrobia-Chlamydiae*)^37^ are exceptional in many aspects, including cell division^38–40^. The *ftsZ* gene is not found in any of the genomes of the *Planctomycetes* and *Chlamydiae*. These bacteria display a variety of division modes, suggesting that divergent mechanisms have evolved within this superphylum. Despite the lack of FtsZ, some planctomycetes, such as the members of the taxa “*Candidatus* Brocadiales” and *Phycisphaerae*, divide by a mechanism similar to binary fission^41^. Others, like the spherical *Gemmata obscuriglobus* and the ovoid *Planctopirus limnophila*, divide by budding^17^. Although the budding mechanism has been studied in depth in yeast cells, the molecular mechanism of bacterial budding remains entirely unknown.

During canonical cell division, FtsZ interacts directly or indirectly with many PG synthesis enzymes. In contrast to other FtsZ-less bacteria, such as the *Tenericutes*, members of the *Planctomycetes* do possess PG^42–45^. Although this polymer was long thought to be absent from *Chlamydiae* and *Planctomycetes*, it was recently detected in some members of these phyla. This experimental detection combined with phylogenomic analyses, suggested that PG is likely to be present in all members of these phyla. It is now clear that Planctomycetes are not exceptions to but deviations from the Gram-negative cell type^46,47^.

Cell division and PG synthesis related genes have been previously detected in members of the *Planctomycetes*^17,18,48,49^. Genomic analyses have revealed that most classically conserved and essential cell division genes show a punctuated pattern of presence in the genomes of *Planctomycetes*. On this basis, we and others have suggested that the divisome and elongasome machineries might be divergent, or even lost, in members of this phylum. Due to their essential role in other bacteria, it was however unclear if these complexes were absent or if they were present but consisted of components that had diverged beyond the point of detection.

Here, we investigate the essentiality of the canonical cell division genes *ftsI, ftsW, ftsK* and *mreB* coding for homologs of proteins that belong to the divisome and elongasome in model bacteria by performing gene knock-outs in the planctomycete *P. limnophila*. We show that the *ftsI, ftsW* and *mreB* genes are not essential in this species.

## Results

We focused here on the *P. limnophila* homologs of the canonical cell division genes *ftsI, ftsW, ftsK* and *mreB*, detected by us and others^17,48,49^, supporting the identification of the homologs. *P. limnophila* FtsI (Uniprot ID: D5SXQ2) is homologous to the *E. coli* protein with 34% identity and similar length: 561 *vs* 572 amino acids (aa). *P. limnophila* FtsW (ID: D5STY0) shares 34% identity with the protein from *E. coli* with similar length, 406 *vs* 414 aa. *P. limnophila* contains only one FtsK homolog (ID: D5SYV8) with 46% identity to *E. coli* protein and size similar to most FtsK homologs (750–900 aa). The MreB homolog (D5SQI2) shares 47% identity to *E. coli* with similar size, 348 *vs* 347 aa, and the corresponding gene seems to be in the same transcriptional unit as *mreC and mreD*, as they are separated by 74 and 3 nucleotides, respectively.

**Figure 1:**
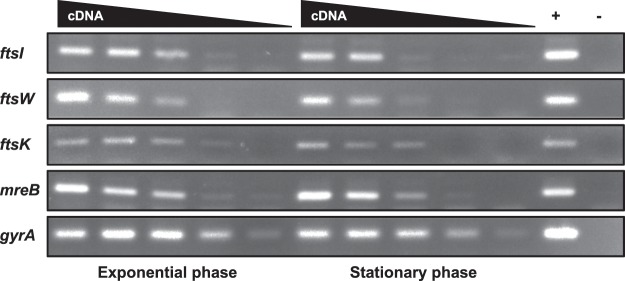
Semi-quantitative RT-PCR of the *ftsI, ftsW, ftsK, mreB* and *gyrA* genes in *Planctopirus limnophila*. Decreasing cDNA template concentration from left to right (125, 25, 5, 1 and 0,2 ng respectively). *gyrA* has been used as housekeeping gene. Positive control: *P. limnophila* genomic DNA, negative control: no DNA. Uncropped image is available in Supplementary Fig. 2.

The chromosome and PG are two important cell components inherited during cell division. FtsK is an ATP-dependent DNA pump, most likely responsible for the transfer of the genetic material from mother to daughter cell. It is highly conserved and is an essential component of the divisome in model bacteria^50^. Despite the punctuated pattern of presence of most of the canonical cell division genes in the planctomycetal genomes, *ftsK* is conserved across the phylum^17^, and semi-quantitative RT-PCR assays showed that it is expressed at the same level throughout the cell cycle, as compared to the control *gyrA* gene, constitutively expressed in most bacteria (Fig. 1). We were unable to generate insertion or deletion mutants of this gene in *P. limnophila*, suggesting that FtsK plays an essential role in this bacterium (identical results were obtained for *G. obscuriglobus ftsK*, supporting the proposed essentiality of the gene; ERM unpublished).

**Figure 2:**
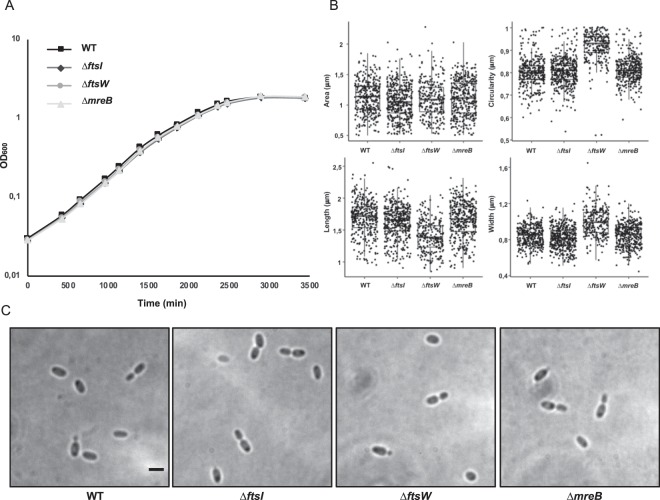
*ftsI*, *ftsW* and *mreB* are non-essential in *Planctopirus limnophila*. (**A**) Growth curves (**B**) morphological measurements (area, circularity, length and width) and (**C**) bright field imaging of the wild type and the *ftsI, ftsW* and *mreB* mutants. Scale bar corresponds to 1 μm. Additional cells for each of the mutants are displayed in Supplementary Fig. 3.

Bacterial cell division is tightly linked to PG synthesis. In model organisms, PG incorporation into the growing PG layer is performed by the divisome at the septum and by the elongasome elsewhere. In the periplasm, the PG precursor is incorporated into the cell wall by various penicillin-binding proteins (PBP). FtsI (PBP3) is one of the PG transpeptidases involved in septal PG synthesis^51,52^. FtsI and FtsW have been shown to interact and to be essential in model bacteria^53^. The genes coding for FtsI and FtsW are conserved in roughly half of the planctomycetal proteomes^17^. In order to decipher if these genes are expressed in *P. limnophila*, we first performed semi-quantitative PCR, demonstrating expression of *ftsI* and *ftsW* in the wild type strain at the same level in exponential and stationary phase (Fig. 1). As suggested by their patchy conservation in *Planctomycetes*^17^, and in contrast to the situation observed in other bacteria, we were able to generate single deletion mutants for both *ftsW* and *ftsI*. Growth curves of the deleted strains did not differ from the wild type (Fig. 2A). Similarly, budding division could not be differentiated from the wild type strain when examined by bright field microscopy (Fig. 2C).

By measuring various cell morphology indexes, the most significant changes were detected for the *ftsW* deletion mutant. The length of the cells of the *ftsW* mutant decreases, while their width increase, resulting in a change of circularity (all with pairwise Wilcox test P value < 10^−15^; Fig. 2B). These changes resulting in slight modifications of the area of the cells (Wilcox test P value > 10^−3^). Nevertheless, as stated above, no differences could be detected in the growth curves or the division modes and thus, the *ftsW* gene is not essential for cell division.

The actin homolog MreB is the main component of the elongasome. It is expected to play a scaffolding role during lateral growth to guide cell-wall elongation in rod-shaped bacteria^30^. Similar to *ftsI* and *ftsW*, *mreB* showed a patchy distribution across the phylum^17^ and we could generate a deletion mutant. Growth curves, morphology and budding phenotype did not differ from the wild type (Fig. 2).

**Figure 3:**
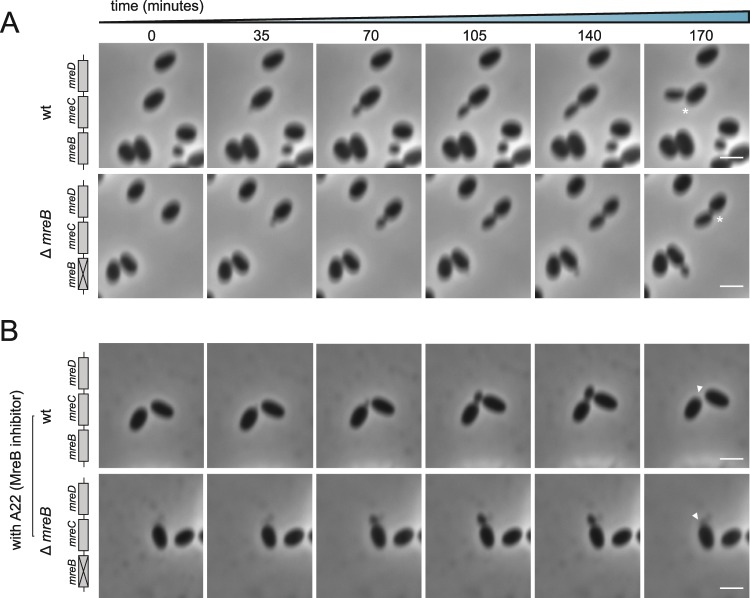
A22 phenotype for the *P. limnophila* wild type and Δ*mreB* mutant. Phase contrast images of still frames of time lapse assays of wild type and Δ*mreB P. limnophila* cells (**A**) without treatment and (**B**) treated with MreB inhibitor A22. Asterisks indicate dividing cells without treatment and arrowheads indicate halted division process by A22 drug. Scale bar corresponds to 1 μm.

The lack of a division phenotype for the Δ*mreB* mutant additionally presented the opportunity to test the effect of the A22 drug in an *mreB*-negative background (Fig. 3). This compound is classically used as an MreB-inhibitor despite the fact that the possible existence of off-target effects has been raised^54^. Before fission, the *P. limnophila* wild type cells increase in cell volume. At the apex of size, the daughter cell bud is formed, and the mother cell decreases in total cell volume. When exposed to A22, wild type and *mreB* deletion mutants, still increased in size (Supp. Fig. S1A) but no longer initiated cell division (Supp. Fig. S1B). These results confirm that, at least in *P. limnophila*, the drug has effects on targets other than MreB, corroborating the proposed unspecific effect of A22.

## Discussion

We report the first evidence of non-essentiality for the divisiome and elongasome components FtsI, FtsW and MreB. This raises the possibility that the divisome and elongasome complexes, essential in the vast majority of bacteria, are absent in some members of the *Planctomycetes* phylum.

Current knowledge about bacterial cell division is predominantly based on model organisms. However, recent studies on various non-model organisms have begun to reveal that some of the statements derived from model organisms do not apply to bacteria universally. A wide variety of different cell division mechanisms is bound to broaden our perspective about bacterial cell division^34,55^.

New evidence has questioned the central role of FtsZ as the predominant force generator during cell division^4,56,57^. An emerging model proposes that assembled FtsZ is a platform to recruit PG synthesis enzymes, and that it is PG synthesis *per se* that directly contributes the forces for septum formation^58^. As all planctomycetes are devoid of FtsZ, this protein cannot be the force generator during cytokinesis in this phylum. However, *P. limnophila* divides by budding, not by binary fission. On the other hand, the planctomycetal class *Phycisphaerae* and order “*Candidatus* Brocadiales” (*i.e*. anammox bacteria) divide by a mechanism similar to binary fission without FtsZ. This raises the question of whether there is a different main protein driving cell division in these organisms.

In *P. limnophila*, *ftsI* and *mreB* deletion do not produce phenotypes. However, we observed that *ftsW* deletion leads to cells that are more spherical compared to the ovoid wild type, without any detectable modification of growth pattern or division mode. FtsW is essential in most organisms containing a single copy of this gene. Depletion of FtsW in rod-shaped bacteria leads to a block in cell division and formation of elongated cells^59–61^. In the polyploid spherical cyanobacterium *Synechocystis* sp. strain PCC 6803, a Δ*ftsW* heteroploid mutant (still retaining wt copies despite deletion of some *ftsW* genes) grows slower and generates giant cells^62^. No such phenotype is observed for the mutants generated in *P. limnophila* which is ovoid and divides by budding.

MreB is essential in model bacteria as well as in bacteria with a divergent division mode, like the longitudinally dividing bacteria of the genus *Thiosymbion*^55^. However, some exceptions exist, such as the ovoid *Streptococcus pneumoniae* and the rod-shaped *Corynebacterium glutamicum*, both devoid of MreB^63^. Many rod-shaped alphaproteobacteria and actinobacteria also lack MreB^64^. On the other hand, some coccoid cyanobacteria contain the *mreB* gene.

*Planctomycetes* belongs to the PVC superphylum that also comprises the *Chlamydiae*, *Verrucomicrobia* and *Lentisphaerae*. The latter two contain FtsZ and divide by binary fission. As for *Planctomycetes*, the chlamydial genomes do not encode for FtsZ and it has recently been reported that a few members of the *Chlamydiae* also exhibit asymmetric, budding-like cell division^35^. In these species, MreB localizes at the cell division site^65^. In most species lacking FtsZ, it is proposed that MreB might substitute for its function^66^. This possibility is revoked here as cells lacking MreB do no display any phenotype.

The non-essentiality of divisome and elongasome genes raises the question of the role of PG itself in *Planctomycetes* and its essentiality to cell integrity and division, despite the importance of these functions in other bacteria. Non-natural forms of PG-deprived cells can be induced in osmoprotective conditions by inhibition of PG synthesis in diverse bacteria^67^. These so-called L-forms divide and can be maintained indefinitely. It has been shown that *ftsZ* becomes non-essential in L-form bacteria, at least in *B. subtilis*^68^. It has been proposed that, in these cell wall-deficient, propagation occurs by an extrusion-resolution mechanism. In addition, the involvement of “some kind of cytoskeletal system” has been suggested and some MreB homologs were directly pinpointed^68^. Interestingly, L-form bacteria appear to be resistant to A22 when grown in the presence of a beta-lactam antibiotic, suggesting that MreB is not required for the growth of spherical L-form-like cells^69^. This is related to our report of A22 susceptibility of the Δ*mreB* mutant which confirms previous suspicion of side-effects of this widely used drug^70^.

Finally, loss of PG synthesis can in some cases lead to non-dividing cells that may enlarge considerably. This has been observed in various species, including *E. coli* and *Vibrio cholera*. Recently it has been shown that giant bacteria could be formed by eliminating essential functions needed for, or treatment with antibiotics targeting PG synthesis in *Acinetobacter baylyi*, a diderm gammaproteobacteria. However, these giant cells did not proliferate^71^. The relevance of these artificially induced cells and division modes and how they compare to *Planctomycetes* division is unclear.

The non-essentiality of the divisome genes for planctomycetal division might appear to be consistent with the previously suggested unusual status of phylum *Planctomycetes* and related PVC superphylum bacteria within the bacterial domain. It seems probable that PVC bacteria are derived from diderm bacteria and that their unknown division modes may have evolved from FtsZ-based binary fission. The deciphering of these alternative division modes and their evolution is important for our sampling of the biodiversity and our understanding of evolutionary cell biology.

Altogether, our results demonstrate that Planctomycetes use divergent division modes, urging for the characterization of the molecular mechanisms of division in *P. limnophila* and in other species of this phylum.

## Material and Methods

### Bacterial strains and culture conditions

*Escherichia coli* DH5α strains were grown in Lysogeny broth medium (LB) at 37 °C and *P. limnophila* DSM 3776^T^ in a modified PYGV medium (DSMZ medium 621 [http://www.dsmz.de]: 0.1% yeast extract, 0.1% peptone, 0.1% glucose, 10 mM HEPES (pH 7,5), vitamin solution and Hutners basal salt solution from DSMZ 590 medium). Planctomycetes were grown at 28 °C. For solid medium, 1.5% bacto-agar was added. To avoid contamination of the planctomycetes when growing for a long time on agar plates, cycloheximide 50 μg ml^−1^ was added. Cultures were grown aerobically in a shaker (180 rpm). When required, antibiotics were used at the following concentrations (μg ml^−1^): kanamycin (Km) 25 for *E. coli* and 50 for *P. limnophila*.

### Plasmid description and genetic modification

Plasmids used for gene deletion in a double event of homologous recombination were derived from the pEX18Tc vector^72^. To construct knockout plasmids for *ftsI, ftsW, ftsK* and *mreB* genes, 1000–1200 bp upstream and downstream fragments of the target gene were amplified by PCR from genomic DNA using the primer pairs listed in Supplementary Table 1. The upstream and downstream fragments were digested with the appropriate enzymes and then cloned into pEX18Tc by three-way ligation. Finally, the kanamycin resistance gene amplified from the pUTminiTn5km plasmid^73^ was subsequently cloned as a *Bam*HI fragment between the two flanking regions. These plasmids enable the deletion of each of the complete genes.

Genetic transformation of *P. limnophila* was performed by electroporation as described before^74^. The cells were then plated onto modified PYGV plates supplemented with kanamycin 50 μg ml^−1^ and were incubated at 28 °C until colony formation (7–9 days). Colonies were transferred to fresh selection plates and genotyped by Southern Blotting and sequencing.

### Mutants sequencing

Deletion mutants were verified by paired-end sequencing on an Illumina MiSeq machine upon library preparation with the Nextera XT DNA Library Prep Kit (Illumina, San Diego, USA). Pre-assembly processing of the reads was done employing Trimmomatic v0.35^75^, FastQ Screen v0.4.4^76^, PRINSEQ lite v 0.20.4^77^ and FLASH v1.2.1^78^. The processed reads were then assembled using SPAdes v3.7.0^79^. Additionally, all reads were mapped to the original sequence of *P. limnophila* with Bowtie 2^80^. The raw sequencing data have been deposited at NCBI SRA under acc. no. PRJNA577131.

### Semi-quantitative RT-PCR

Total RNA extraction was carried out as previously described^81^ from 20 ml of wild type cultures at exponential (OD_600_ ~0,4) and stationary phase (OD_600_ ~1,5). DNase I treatment was performed with a DNA-free kit (Ambion). The samples were purified using RNAeasy columns (Quiagen) and RNA quality was confirmed by non-denaturing agarose gel electrophoresis. The absence of contaminating DNA was then confirmed by PCR amplification.

Reverse transcription of the RNA samples was performed using the High-Capacity cDNA Archive Kit (Applied Biosystems), with random hexamers as primers to generate cDNAs. The resulting cDNA samples were amplified by semi-quantitative RT-PCR using 1 mM of each primer (Supplementary Table 2). Genomic DNA was used as a positive control of the PCR. Samples were visualized in 10% acrylamide gels.

### Microscopy

Bacteria from 2 ml of exponentially growing culture (OD_600_ ~0.4) were harvested (12000 *g*, 3 min) and resuspended in 100 μl of fresh medium. A sample of 2 μl was spotted on a glass-bottom dish (MatTek) and covered with a 1% agarose in M3 medium cushion, as described by^82^. Bright field images were acquired using a 100×/1.46 objective through an 1.6X amplification lens and an EMCCD Andor iXon camera mounted on a Zeiss microscope, resulting in a pixel size of 0.1 × 0.1 µm.

### Image analysis

An automatic analysis workflow was design using FIJI until a satisfactory segmentation of the cells was achieved. The image analysis workflow runs as follows under FIJI software: Image Acquisition → Subtract Background → Gaussian Blur → Invert → Enhance Contrast → Unsharp Mask → Watershed Thresholding → Convert to mask → Binary Watershed → Analyze Particles (with a size upper and lower limit of 21 ∙ 10^−3^ to 3.5 ∙ 10^−3^ µm. Any particle out of this limit was not considered as a cell. Afterwards, the area of the cells was measured and fitted to an ellipse. In order to extract cell’s width and length, we identified them as the ellipse’s minor and major axis, calculating subsequently the circularity. The values of each single cell were exported for further statistical analysis with R.

### A22 inhibition assay

Exponentially growing cultures (OD_600_ ~0.4) were loaded on a CellASIC ONIX plate for bacteria (EMD Millipore). The plate was then flushed with fresh medium without A22 for at least 12 hours to get the cells accustomed to their new environment. The plate was then flushed with fresh medium constituted with A22 (20 μg ml^−1^). Images were acquired every five minutes using a Leica DMi8 microscope with a 1000x phase contrast objective using an 8 micron Z-stack.

### Statistical methods

All statistical analyses were done with R with >250 observations for each features. Normality was tested with the Bartlett test. The identity of the distributions was evaluated with the kruskal test, and the groups compared pairwise with the Wilcox test for non-parametric statistical tests.

## Supplementary information

Supplementary Information 
