# Supplementary material for: Non-essentiality of canonical cell division genes in the planctomycete Planctopirus limnophila
Source: Sci Rep. 2020 Jan 9;10:66. doi: 10.1038/s41598-019-56978-8 (PMC6952346; doi:10.1038/s41598-019-56978-8)
Supplement: Supplementary file 1 — Supplementary Information [file 41598_2019_56978_MOESM1_ESM.pdf]

Non-essentiality of the canonical division genes in the planctomycete *Planctopirus limnophila*

Elena Rivas-Marin[1], Stijn H. Peeters[2], Laura Claret Fernández[1, 2], Christian Jogler[2,3], Laura van Niftrik[2], Sandra Wiegand[2], Damien P. Devos[1]

[1]Centro Andaluz de Biología del Desarrollo (CABD)-CSIC, Pablo de Olavide University, Seville, Spain.

[2]Department of Microbiology, IWWR, Faculty of Science, Radboud University, Nijmegen, The Netherlands.

[3]Institute of Microbiology, Department of Microbial Interactions, Friedrich-Schiller-Universität Jena, Germany.

Supplementary material:

3 Supplementary Figures.

2 Supplementary Tables.

Supplementary Figure 1. A) The *Planctopirus limnophila* cells affected by MreB inhibitor A22 no longer divide and start gaining cell volume. Cell cycles of cells not affected by A22 gain volume and start the fission process when the volume lost during the precious cell division has been regained. Phase I: the preparation phase. Phase II: Size increase of mother cell. Phase III, bud formation and increase of bud cell volume, and simultaneous decrease of mother cell volume. Phase IV: phase affected by MreB inhibitor A22, where daughter and mother cell volume increase asymptotically, but no bud formation occurs. B) Cell division events over time cell over time with and without A22 for the MreB deletion mutant (red) and the wild type (black). Each vertical line indicates a single cell division initiation.

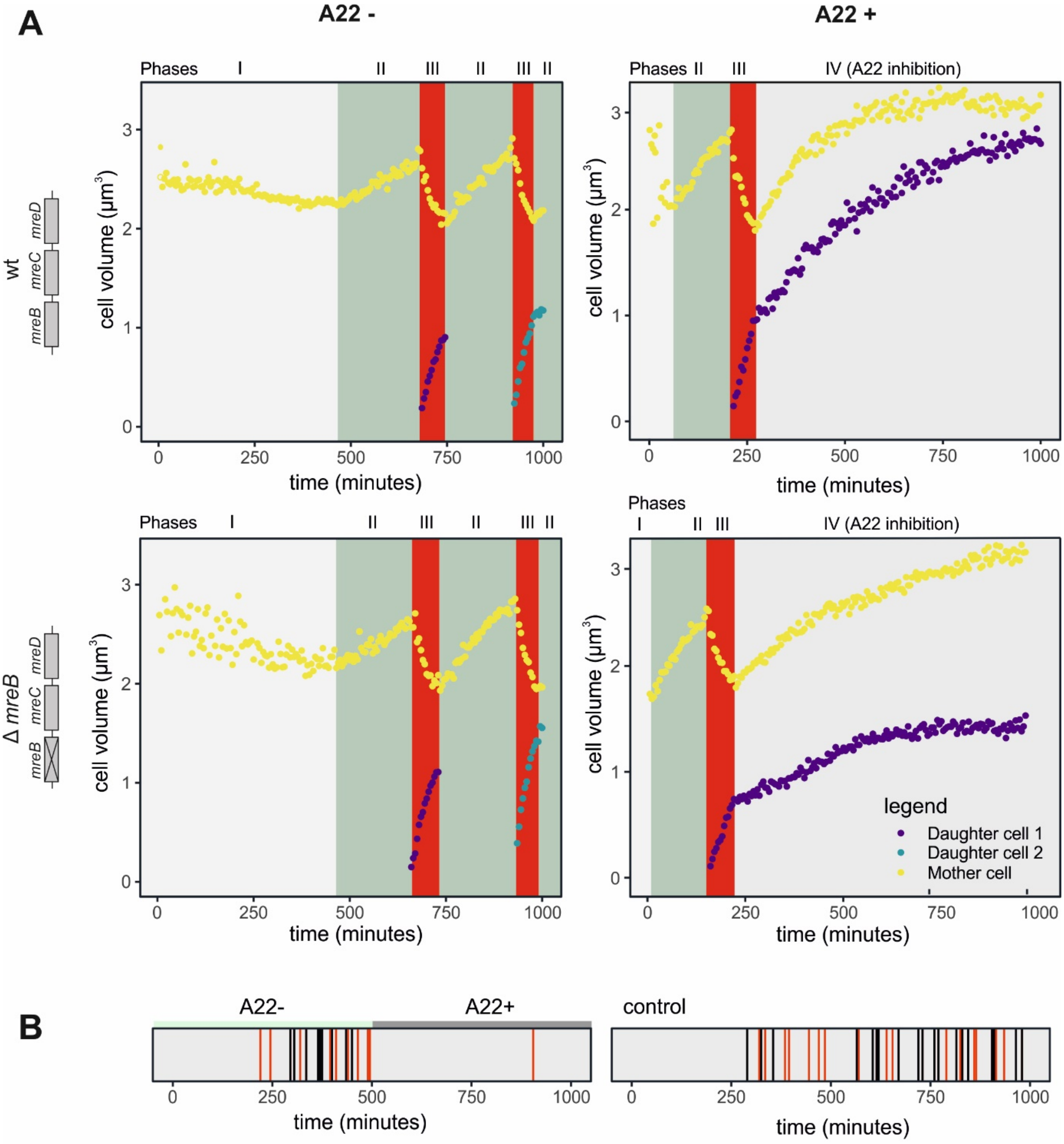

Supplementary Figure 2. Uncropped image of Figure 1 of the main text.

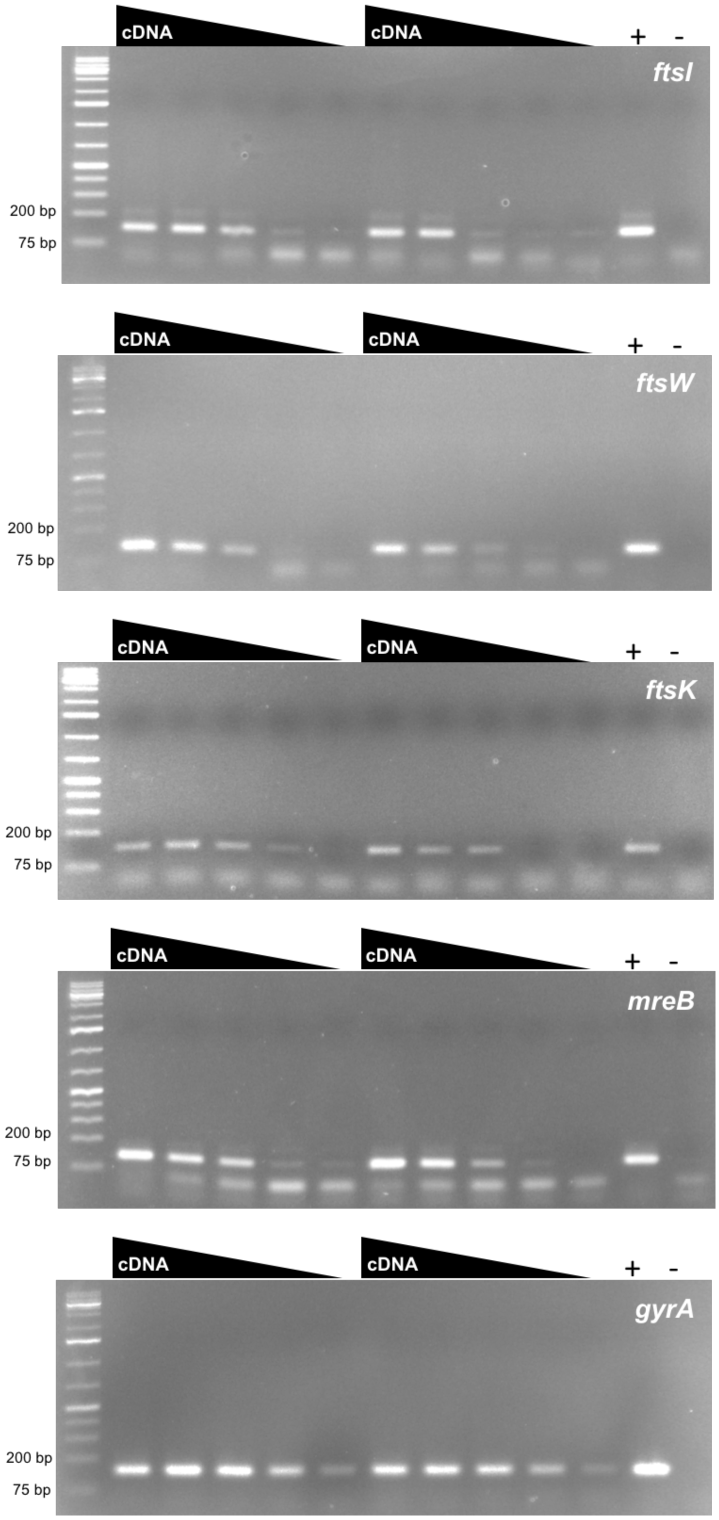

Supplementary Figure 3. Overview of bright field imaging of the A) wild type, B) Δ*ftsI*, C) Δ*ftsW* and D) Δ*mreB Planctopirus limnophila* mutants. Scale bar corresponds to 1μm.

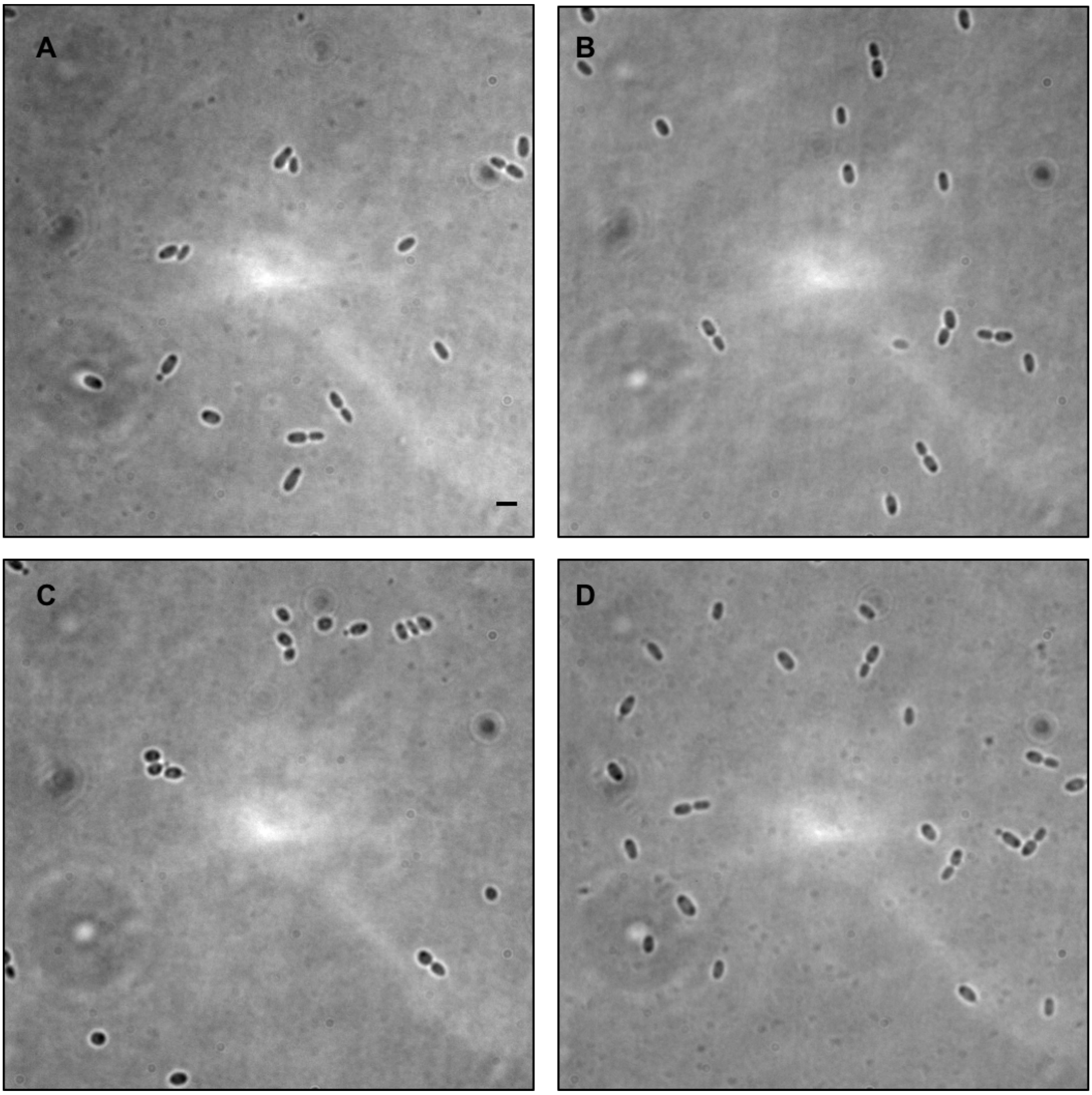

Supplementary Table 1: oligonucleotides used for cloning purpose. Underlined restriction sites.

**Primer name**	**Primer sequence**	**Target**
Left ftsI limno fwd	GTAGAATTCATCCAGATTCTCGAACTCAAC	*ftsI* upstream region
Left ftsI limno rv	TTGGGATCCCGCTGCTCCATCGAACGAG	*ftsI* upstream region
Right ftsI limno fwd	GTAGGATCCGAGCTTTCACGCAGGATTTC	*ftsI* downstream region
Right ftsI limno rv	TTGGTCGACGCTGACGGAATGAGCGTCG	*ftsI* downstream region
Left ftsW limno fwd	GTAGAATTCCCAGAACATCGACATG	*ftsW* upstream region
Left ftsW limno rv	TTGGGATCCTCCCCTCGGCTCCACATTTC	*ftsW* upstream region
Right ftsW limno fwd	TTGGGATCCGTCTGACGATCTTGAGGACTG	*ftsW* downstream region
Right ftsW limno rv	GTAAAGCTTGGCAAACAAAACAGCCGATGG	*ftsW* downstream region
Left ftsK limno fwd	GACCTTAAGCGTCCGTCGG	*ftsK* upstream region
Left ftsK limno rv	GTAGGATCCCTAGAGCAGCAGAATCCATGCTC	*ftsK* upstream region
Right ftsK limno fwd	GTAGGATCCCTTCCACAGGCAATCGACC	*ftsK* downstream region
Right ftsK limno rv	TTGAAGCTTTTCGCCAATCAGCACGAGTG	*ftsK* downstream region
Left mreB limno fwd	GTATCTAGATCCACCGACGCACGAATAC	*mreB* upstream region
Left mreB limno rv	TTGGGATCCGATTCACTTTGCGAACGATATC	*mreB* upstream region
Right mreB limno fwd	TTGGGATCCGACAGGGGCTGCTCTTTAC	*mreB* downstream region
Right mreB limno rv	GTAGTCGACAGGATAGCAAACGCTTCATGG	*mreB* downstream region
Km BamHI fwd	GTTGGATCCGCGTCGGCTTGAACGAATTG	Kanamycin resistant gene

Km BamHI rv	TGAGGATCCCATTTCGAACCCCAGAGTCC	Kanamycin resistant gene

Supplementary Table 2: oligonucleotides used for semi-quantitative RT-PCR assays in *P. limnophila*.

**Primer name**	**Primer sequence**	**Target**
FtsI sq 2 fwd	CGAACAGCCTCGGGACAGC	*ftsI*
FtsI sq 2 rv	CATCAATCAGGCTGGGAACC	*ftsI*
FtsW sq fwd	CCCGGAAATCCTGGAACTTAAG	*ftsW*
FtsW sq 2 rv	AGAGCCCGAAACCTAGCAGC	*ftsW*
FtsK sq fwd	TCTCACCTTGTGCATCCTGCT	*ftsK*
FtsK sq 3 rv	TCGATCACCACGAGCATGAG	*ftsK*
SemiQ mreB fwd	TCCACAGGTTACGGCAGTGG	*mreB*
SemiQ mreB fwd	CTGAAGAGCGACGACTGTGG	*mreB*
SemiQ gyrA fwd	GAAATCTCCTCTGAGACCCG	*gyrA*
SemiQ gyrA rv	ATCAGCCGCAATTCTTCGTAC	*gyrA*
